# Single-dose pharmacokinetics and lung function of nebulized niclosamide ethanolamine in sheep

**DOI:** 10.1007/s11095-023-03559-0

**Published:** 2023-07-27

**Authors:** Anne Weiss, Robert J Bischof, Cornelia B Landersdorfer, Tri-Hung Nguyen, Andrew Davies, Jibriil Ibrahim, Paul Wynne, Phillip Wright, Günter Ditzinger, A Bruce Montgomery, Els Meeusen, Michelle P McIntosh, Morten OA Sommer

**Affiliations:** 1 UNION therapeutics Research Services, Hellerup, Denmark; 2 Novo Nordisk Foundation Center for Biosustainability, Technical University Denmark, Lyngby, Denmark; 3 Allergenix Pty Ltd, Melbourne, Australia; 4 Institute of Innovation, Science and Sustainability, Federation University Australia, Berwick, Australia; 5 Drug Delivery, Disposition and Dynamics, Monash University, Melbourne, Australia; 6 Biomedicine Discovery Institute, Monash University Peninsula Campus, Frankston, Australia; 7 UNION therapeutics, Hellerup, Denmark; 8 Medina, Washington, USA

**Keywords:** Niclosamide, nebulization, pulmonary pharmacokinetics

## Abstract

**Purpose:**

Niclosamide is approved as an oral anthelminthic, but its low oral bioavailability hinders its medical use requiring high drug exposure outside the gastrointestinal tract. An optimized solution of niclosamide for nebulization and intranasal administration using the ethanolamine salt has been developed and tested in a Phase 1 trial. In this study we investigate the pulmonary exposure of niclosamide following administration via intravenous injection, oral administration or nebulization.

**Methods:**

We characterized the plasma and pulmonary pharmacokinetics of three ascending doses of nebulized niclosamide in sheep, compare it to intravenous niclosamide for compartmental PK modelling, and to the human equivalent approved 2 g oral dose to investigate in the pulmonary exposure of different niclosamide delivery routes. Following a single-dose administration to five sheep, niclosamide concentrations were determined in plasma and epithelial lining fluid (ELF). Non-compartmental and compartmental modeling was used to characterize pharmacokinetic profiles. Lung function tests were performed in all dose groups.

**Results:**

Administration of all niclosamide doses were well tolerated with no adverse changes in lung function tests. Plasma pharmacokinetics of nebulized niclosamide behaved dose-linear and was described by a 3-compartmental model estimating an absolute bioavailability of 86%. ELF peak concentration and area under the curve was 578 times and 71 times higher with nebulization of niclosamide relative to administration of oral niclosamide*.*

**Conclusions:**

Single local pulmonary administration of niclosamide via nebulization was well tolerated in sheep and resulted in substantially higher peak ELF concentration compared to the human equivalent oral 2 g dose.

**Supplementary Information:**

The online version contains supplementary material available at 10.1007/s11095-023-03559-0.

## Introduction

Niclosamide is a salicylanilide introduced in the 1960s as oral chewable tablets to treat tapeworm infections [1, 2]. Niclosamide has been subject to many repurposing studies demonstrating therapeutically relevant activities across a wide range of indications ranging from metabolic diseases, autoimmune diseases and, cancer to pulmonary conditions and infections [3].

The major challenge with repurposing niclosamide using the oral chewable tablets for these indications is its low oral bioavailability and poor solubility, and rapid metabolism in the liver which limits systemic absorption and distribution to tissues outside the gastrointestinal tract and excretory organs [4, 5]. The oral bioavailability has been reported as 5% - 10% in rats and 0.5% in dogs [6, 7]. Furthermore, two clinical trials using oral niclosamide in a few adult cancer patients reported variable systemic concentrations ranging from 665-759 ng/mL (5 patients receiving 2 g/day) and 35.7-182 ng/mL (3 patients receiving 1.5g/day) with dose limiting toxicities being observed when higher doses than the approved 2 g/day were used [8, 9]. Recently published data from a Phase 2 trial with oral niclosamide in patients with mild-moderate COVID-19 did not meet its primary endpoints using the approved 2 g oral dose, although there was a trend towards reduced viral loads in fecal shedders of the active arm [10]. This study is consistent with the hypothesis that the effects of niclosamide, when administered orally as a 2 g dose, is restricted to the gastrointestinal tract, highlighting the need for novel delivery approaches for indications with pathology beyond the gastrointestinal system.

To explore the potential of niclosamide for treatment of infections of the lower and upper respiratory system, local delivery may be preferred over other routes if a high local concentration can be achieved while limiting systemic exposure and toxicity. Several groups have attempted the reformulation of niclosamide for pulmonary administration. Brunaugh *et al.* developed a dry powder formulation of niclosamide by encapsulating niclosamide in human-lysozyme and showed improved survival and significant reduction in lung viral titers when administered intranasally to SARS-CoV-2 and MERS-CoV infected mice [11]. A subsequent pharmacokinetic (PK) study by Jara *et al*. in which the same formulation was administered via inhalation in rats (~0.8 mg/kg) and hamsters (0.14-0.29 mg/kg) found peak concentrations of 20-205 ng/g and 20.5-37.2 μg/g in lung tissue, respectively [12]. This dry powder formulation has progressed to clinical trials, with a completed Phase 1 trial in healthy volunteers showing that inhalation of 0.5, 2 and 6 mg niclosamide was well tolerated, with no dose limiting cough or irritation observed [13]. No lung pharmacokinetics were reported. Costabile *et al*. administered a niclosamide nanosuspension (10 - 100μg) intratracheally to Wistar rats and found no signs of acute toxicity or lung injury despite mild to moderate infiltration of macrophages and neutrophils in the bronchoalveolar lavage fluid at the highest dose [14], that may have been mediated by exposure to niclosamide or vehicle.

UNION therapeutics has developed an optimized solution of niclosamide for nebulization and intranasal administration using the ethanolamine salt which has been tested and found to be well tolerated in a Phase 1 trial in healthy volunteers [15].

Here we show the pharmacokinetics of niclosamide in plasma and epithelial lining fluid (ELF) coupled with lung function assessment in sheep, a recognized large animal model with a pulmonary anatomy similar to humans [16–18], following nebulization of a 0.167%, 0.5% and 1% niclosamide ethanolamine (NEN) solution. Nebulized NEN doses were chosen to reflect the dose ranges used in the Phase 1 trial [15]. Nebulized NEN was compared to intravenous NEN for development of a compartmental population pharmacokinetic model to determine nebulized bioavailability, and oral niclosamide to compare niclosamide ELF concentrations following nebulized NEN to the human equivalent approved 2 g oral dose.

## Materials and Methods

### Animals

Merino cross-bred female sheep (n=10 ewes) used in this study were sourced from a commercial livestock supplier. All sheep were treated orally with anthelminthic to eliminate any worm parasites (in line with standard management practices) and received standard flock health vaccinations prior to delivery to the Monash Animal Research Platform Facility at Monash University. Upon arrival, sheep were acclimatized for at least 5 days before study commencement, rotated between small group pen housing and metabolic cages under controlled ambient conditions (20-22°C) and maintained on a 12 h light/dark cycle throughout the experimental period. For nebulization administration and bronchoalveolar lavage fluid (BALF) sampling, sheep were positioned in a specialized harness to restrict movement of head and neck. An intravenous catheter (Angiocath 16 g 3.25 in, Becton Dickinson) was inserted into the jugular vein prior to the commencement of experimentation, was maintained on a daily basis and used throughout the duration of the experimental period. All sheep were 12 months of age and had a weight of 25-35 kg. The animal study received approval from the Federation University Australia/Monash University animal ethics committees (19-007).

### Niclosamide Preparations and Dosing Schedule

Niclosamide was administered via nebulization, intravenous (IV) and oral route of delivery. Three different formulation strengths were used for nebulization: 0.167 %, 0.5 % and 1 % NEN solution (w/v) as well as a placebo solution containing only the vehicle of the 1% NEN solution. A 0.1% NEN solution was used for IV administration (10-fold dilution of the 1% NEN solution in saline prior to administration). Niclosamide for oral dosing was prepared by crushing the commercially available tablet formulation (Yomesan®, 500 mg tablet, Bayer [2]) and dispersing it in 20 mL sterile water to a final dose similar to the approved oral 2 g human dose on a mg/kg and mg/m^2^ basis (Table I).

**Table I:** Dose and administration route per dose group and corresponding sampling times of BALF and blood samples

Experiment (n = 5)	Dose Group	Administration route Dose	Sampling times BALF	Sampling times Blood
1 (Sheep # 1 -5)	1	Nebulization 1% Placebo Solution	-	-
	2	Nebulization 6mL, 0.167% NEN 10 mg NEN	- 0 h (predose) - 5 min, 30 min, 1 h, 4 h, 8 h (after completion of dosing)	- 0 h (predose) - 7.5 min, 15 min (during dosing) - 0, 5 min, 15 min, 30 min, 1 h, 2 h, 3 h, 4 h, 6 h, 8 h, 12 h (after completion of dosing)
	3	Nebulization 6mL, 0.5% NEN 30 mg NEN		
	4	Nebulization 6mL, 1% NEN 60 mg NEN		
2 (Sheep # 11-14,18)	5	Oral 1132 mg^1^		- 0 h (predose) -0, 5 min, 15 min, 30 min, 1 h, 2 h, 3 h, 4 h, 6 h, 8 h, 12 h (after completion of dosing)
	6	Intravenous 3mL, 0.1% NEN 3 mg NEN		- 0 h (predose) - 7.5 min, 15 min (during dosing) - 0, 5 min, 15 min, 30 min, 1 h, 2 h, 3 h, 4 h, 6 h, 8 h, 12 h (after completion of dosing)

NEN = Niclosamide ethanolamine.

^1^ corresponds to the human equivalent dose of the 2g human oral dose based on a mg/kg and a mg/m^2^ basis, assuming a 60 kg human weight and 34 kg sheep weight. For the mg/m^2^ conversion a k_m_ of 32 and 37 was used for sheep and human, respectively (according to FDA guidance for Industry: Estimating the Maximum Safe Starting Dose in Initial Clinical Trials for Therapeutics in Adult Healthy Volunteers). The actual delivered dose was 1132 mg niclosamide (mean) as on average 27.88 ± 1.64 mg/kg were delivered to a 40.6 ± 2.1 kg sheep.

All sheep were fasted for 12-24 h prior to drug treatment. For each dose group, a single dose was administered followed by a 7 day “wash out” period prior to the next dose (see Table I for order of doses). In Experiment 1, five sheep were allocated to dose group 1-4 in an ascending dose manner. In Experiment 2, a separate cohort of 5 sheep were allocated to dose group 5 and 6 where a single dose was also administered with a 7 day “wash-out” period (Table I). The IV arm was included to understand the absolute bioavailability of niclosamide and the oral arm to compare exposure in the ELF following oral administration versus the nebulized route.

### Niclosamide administration procedures

All dosing procedures were performed while experimental subjects were fully awake, and judged to be in good health. The actual delivered dose (mg/kg), delivery time and droplet size distribution, where applicable, are summarized for each dose group in Table S1 (supplementary information).

For nebulized niclosamide dosing, aerosol delivery was performed as previously described in a closed respiratory loop [16, 17]. Briefly, a lubricated cuffed endotracheal tube (Portex, 7.0-8.00 mm internal diameter) was inserted via the nasal passage into the trachea and attached to a dual phase control respirator (Harvard Apparatus, MA, USA) (20 breaths/min, 50% inspiration). Filters (Hudson, RCI, NC, USA) were placed in the expiratory line, and together with all intubation lines and nebulizer components, were removed following completion of dosing and rinsed with 50 mL MilliQ Water to determine any residual drug and allow calculation of the actual dose. The three different NEN solutions (6 mL) were administered via the investigational eFlow Inline Nebulizer manufactured by PARI Pharma GmbH. The niclosamide aerosols generated from the nebulizers exhibited a D_50_ droplet size (Malvern Spraytec, Malvern PanAnalytical, Bristol, UK) of 3.38 μm ± 0.26 μm and were delivered within a mean timeframe of 24-27 min per dosing treatment. The actual delivered dose (mg/kg), nebulization time and droplet size distribution are summarized for each dose group in Table S1 (supplementary information).

For oral niclosamide delivery, an established procedure [19, 20] (with slight modifications) was used for closure of the reticular groove in sheep to enable oral drug delivery directly into the abomasum or true stomach. Briefly, this involved oral delivery of 10% CuSO_4_ (w/v) solution (in 20 mL sterile water) to stimulate closure of the reticular groove, via a feeding tube (7 mm internal diameter) that was inserted through the nasal passage into the esophagus. CuSO_4_ was immediately followed by delivery of the niclosamide suspension made from crushed tablets (in 20 mL sterile water) followed by a glucose ‘chaser’ (300 mL glucose solution (25% w/v)). The glucose solution allowed verification that each oral dose was successfully delivered to the abomasum (true stomach) and subsequently absorbed via the intestine (blood glucose concentrations measured at 15 min and 30 min post-dosing confirmed direct delivery into the abomasum; see supplementary information, Fig. S1).

For IV administration, 3 mL of a 0.1% NEN solution was administered via an infusion pump and IV catheter (inserted into the jugular vein) at a rate of 0.1 mL/min for 30 min.

### Lung function assessments

Lung function measurements were undertaken in awake, consciously breathing sheep, immediately after and again at 24 h following each dosing (for up to 10 min on each occasion), according to established protocols [16]. Lung parameters were derived from averaged measures of five measured epochs of five breaths and data analyzed using LabChart™ software. Comparison of lung function parameters pre – and postdosing were compared using paired Student’s T-test.

### Sampling

Peripheral blood and BALF samples were obtained from all sheep in all dose groups except the placebo group according to the sampling schedule in Table I. Peripheral blood samples (14 time-points over 12 h) were collected from a cannulated jugular vein into K_3_EDTA containers (BD Biosciences), and plasma samples stored at -80°C until analysis. BALF samples (6 time-points) were collected by intra-lung infusion of 25 mL saline via a catheter through the biopsy port of the bronchoscope into separate lung segments/lobes: 0 h (right apical lobe), 5 min (left middle), 30 min (right caudal), 1 h (left caudal), 4 h (right middle), 8 h (left apical) [16, 17]. Cell-free BALF samples were stored at -80°C until analysis. Mean BALF recovery was 9.28 ± 1.72 mL with no significant differences across the 6 sampled lobes. Blood and BALF sampling times were overlapping to allow back calculation of ELF dilution to estimate drug concentration in the ELF using the urea correction method [18]. Urea concentrations were measured in BALF and plasma samples using a commercial plate-based assay according to manufacturers’ instructions (LTS-EIA-BUN, Thermo Fisher, Waltham USA). Actual collection times were recorded for compartmental population pharmacokinetic modelling.

### Quantification of Niclosamide in Biological Samples

Niclosamide was quantified in plasma and BALF using a validated liquid chromatography-mass spectrometry method (Shimadzu 8050 triple quadrupole instrument coupled with Shimadzu Nexera X2 UHPLC). Linearity was confirmed in the range of 0.5 ng/mL – 500 ng/mL for plasma samples and 0.5 – 1000 ng/mL for BALF samples. One BALF sample was diluted 1:1 with blank BALF to bring it into the range of the standard curve. The niclosamide concentrations in ELF were estimated according to the urea correction as previously described by Landersdorfer *et al*. [18].

### Non-compartmental and compartmental Pharmacokinetic analysis

PKSolver add-in for Excel was used for non-compartmental analysis of plasma pharmacokinetic data. Due to variable and sparse ELF concentrations, ELF AUCs were calculated using trapezoidal calculations in excel. GraphPad Prism Version 9.1.2. was used for visualization.

Compartmental population pharmacokinetic (PK) modelling of niclosamide following IV and nebulized dosing was conducted via non-linear mixed-effects modelling. The first-order conditional estimation with interaction (FOCE+I) algorithm in NONMEM® Version 7.2 (ICON Development Solutions, Ellicott City, MD) was utilized for modelling. R scripts were utilized for generation of model evaluation plots using RStudio Version 1.1.463 (RStudio, Inc.).

Models containing one, two and three disposition compartments were evaluated. Niclosamide concentrations in plasma from all sheep and all dose levels were modelled simultaneously. Models incorporating linear, non-linear and parallel linear and non-linear elimination were evaluated. The systemic bioavailability of niclosamide following nebulized administration was estimated, assuming that the systemic elimination and distribution of niclosamide were similar for IV and nebulized dosing. Models including different absorption rate constants and/or different extents of bioavailability following nebulized dosing for the different dose levels were tested. A log-normal distribution was used to describe the interindividual variability of the PK estimates. Models with and without covariance between clearance and volume of distribution were evaluated. The residual unexplained variability for niclosamide in plasma was described by a combined proportional and additive error model.

For model evaluation, plots of observed versus individual-fitted and observed versus population-fitted niclosamide concentrations (supplementary information, Fig. S2), conditional weighted residuals, the normalized prediction distribution error and the NONMEM objective function were utilized. For each model evaluated, NONMEM computed the minimum value of the objective function, a statistic equivalent to minus twice the log likelihood (-2LL). The log-likelihood ratio was used to compare two nested (hierarchical) models, and Δ−2LL was the change in −2LL (i.e. the NONMEM objective function value) relative to the comparator model. The best-fit model displayed the lowest objective function value. A reduction in the objective function by more than the critical value of 3.84 units (Δdf =1 for one added degree of freedom) represented a statistically significant improvement in model fit (chi^2^ distribution, p<0.05). In addition to the statistical model selection criteria, the plausibility of the parameter estimates was considered.

The final model to describe the plasma PK following IV and nebulized dosing simultaneously was a 3-compartment disposition model with linear (non-saturable) elimination clearance and linear (first-order) absorption, with interindividual variability on clearance, central volume of distribution, volume of distribution of the shallow peripheral compartment and the absorption rate constant. The model well described the observed concentrations across all sheep and dose levels and both routes of administration.

The amount of niclosamide in the absorption compartment (A_abs_) was described as:

$$\frac{dA_{abs}}{dt}={Input}_{neb}\hbox{--} {k}_{\alpha}\bullet {A}_{abs}$$

where Inputneb was the dose administration *via* nebulization modelled as a time-delimited zero-order, *i.e.* constant-rate, input and ka was the first-order absorption rate constant from the absorption compartment to the systemic circulation.

The amount of niclosamide in the central compartment (A1) was described as:

$$\frac{\textrm{dA}1}{\textrm{dt}}=\kern0.5em {k}_{\alpha}\bullet {F}_{neb}\bullet {A}_{abs}+{Input}_{IV}- CL\bullet C1\hbox{--} {CL}_{d1}\bullet C1+{CL}_{d1}\bullet C2-{CL}_{d2}\bullet C1+{CL}_{d2}\bullet C3$$

where F_neb_ was the bioavailability following nebulized dosing, Input_IV_ was the dose administration via IV infusion that was modelled as a time-delimited zero-order, i.e. constant-rate, input, CL was the systemic elimination clearance, C1 was the concentration in the central compartment, CL_d1_ was the distribution clearance between the central and the shallow peripheral compartment, C2 was the concentration in the shallow peripheral compartment, CL_d2_ was the distribution clearance between the central and the deep peripheral compartment and C3 was the concentration in the deep peripheral compartment.

The amount of niclosamide in the shallow peripheral compartment (A2) was described as

$$\frac{\textrm{dA}2}{\textrm{dt}}=\kern0.5em {CL}_{d1}\bullet C1\hbox{--} {CL}_{d1}\bullet C2$$

The amount of niclosamide in the deep peripheral compartment (A3) was described as

$$\frac{\textrm{dA}3}{\textrm{dt}}=\kern0.5em {CL}_{d2}\bullet C1\hbox{--} {CL}_{d2}\bullet C3$$

## Results

### Lung function tests following nebulized, IV and oral niclosamide

**Fig. 1:**
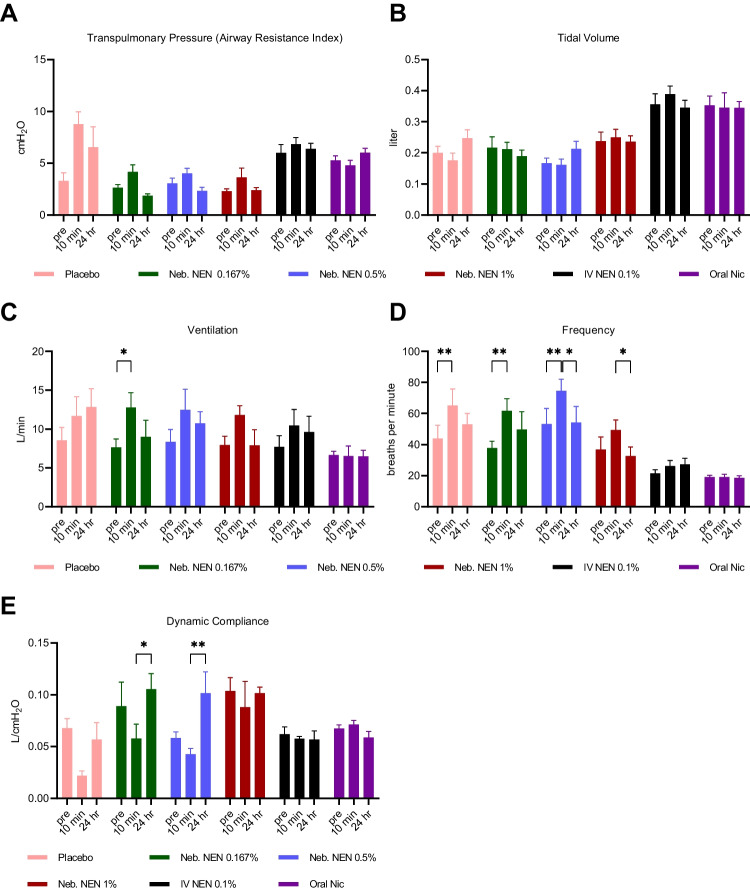
Lung function assessment before and after administration of nebulized placebo, nebulized and intravenous niclosamide ethanolamine, and oral niclosamide. **A** Transpulmonary pressure, **B** Tidal volume, **C** Ventilation, **D** Breathing frequency, **E** Dynamic compliance. Data from n=5 sheep. Mean ± SD displayed. * p< 0.05, ** p < 0.01. Neb. = Nebulized, NEN = niclosamide ethanolamine, Nic = Niclosamide, IV = intravenous. Lung function assessment was performed at 5 min and not 10 min postdosing for the 0.167% NEN and 0.5% NEN treatment arm.

Lung function parameters were assessed in 5 animals before (pre-) and after (post-) nebulized, IV and oral niclosamide administration (Fig. 1). Following nebulized administration, no significant changes in transpulmonary pressures (airway resistance) or tidal volumes were observed. There was a transient increase in ventilation and breathing frequency seen in the period immediately following dosing (Fig. 1C and D), which likely led to the brief shift observed in the dynamic compliance index (Fig. 1E). For the IV and oral doses, no significant difference was observed in mean transpulmonary pressures (airway resistance) or dynamic compliance before (pre-) and after (post-) dose administration. Across all dose groups, niclosamide was well tolerated as no adverse signs were observed.

### Plasma Pharmacokinetics of niclosamide after nebulized administration

**Fig. 2:**
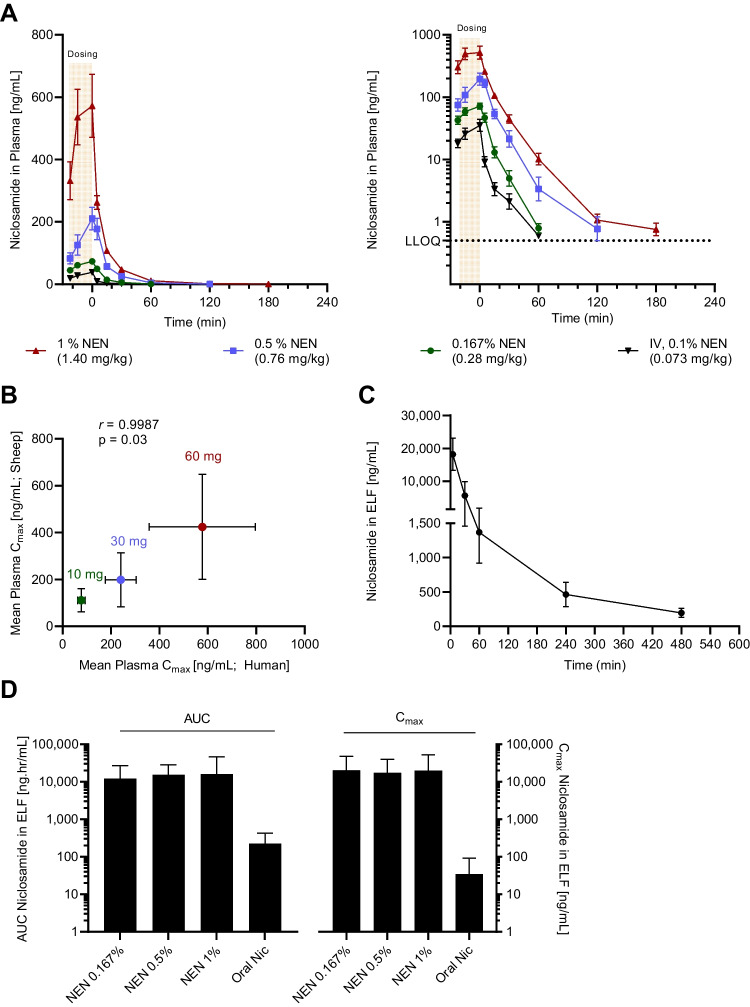
Pharmacokinetics of niclosamide following nebulization in sheep. **A** Time-course profile of plasma concentration of niclosamide following nebulization of 0.167%, 0.5% and 1% niclosamide ethanolamine (NEN) solution and 0.1% NEN solution via intravenous administration on linear (left panel) and semilog scale (right panel). Eight datapoints from individual sheep were excluded in non-compartmental analysis and are not shown here as they could not be explained by protocol deviation and disagreed with basic pharmacokinetic principles but are displayed in Fig. S4 in the supplementary information. **B** Pearson correlation of mean plasma C_max_ of the three nebulized doses of this study to mean plasma C_max_ of dose-matched cohorts of Phase 1 trial by Backer *et al.* [15]*. r* = pearson correlation coefficient. Mean ± SEM displayed. **C** Time-course profile of niclosamide concentration in epithelial lining fluid (ELF) as combined profile following 0.167% ,0.5% and 1% NEN nebulized administration on linear scale. **D** Area-under the curve (AUC) and maximum concentration (C_max_) of niclosamide in ELF following nebulized NEN versus the human equivalent 2 g oral dose (corresponding to 1132 mg niclosamide). For panel **A**, **C**, **D** mean ± SEM is displayed, and data is from n=5 sheep per group. LLOQ = lower limit of quantification.

Mean single doses of 0.27 mg/kg (0.167%), 0.76 mg/kg (0.5%) and 1.40 mg/kg (1%) NEN were administered nebulized over 24 to 27 min. Following nebulization of NEN, the apparent plasma C_max_ of niclosamide was reached immediately post-dosing in all three dose strengths with peak concentrations of 76.7, 239.8 and 577.1 ng/mL, respectively. Thereafter, there was a rapid decline in niclosamide concentrations over the first 2 h post-dose for all three dose strengths, with numerous plasma concentrations reaching levels close or below the LLOQ of 0.5 ng/mL at 1 to 2 h post-dose (Fig. 2A). Plasma pharmacokinetics of niclosamide following a single intravenous infusion of 0.1% NEN, which was used to estimate nebulized bioavailability in the compartmental model, followed similar kinetics as the 0.167% nebulized dose reaching low plasma concentrations at 1 h post-dose (Fig. 2A).

The plasma pharmacokinetic profile of niclosamide was characterized by short half-lives in the alpha- and beta-phases (0.041 h and 0.2 h, respectively) and a substantially longer elimination half-life of 14 h.

The final population pharmacokinetic model describing the plasma pharmacokinetics following IV and nebulized dosing simultaneously was a 3-compartment disposition model with linear (non-saturable) elimination clearance and linear (first-order) absorption (see goodness of fit plots in Fig. S2 in supplementary information). The population mean bioavailability of nebulized NEN was estimated to be high at 86% (Table II). Furthermore, all modelling results indicated linearity in absorption and systemic plasma pharmacokinetics of niclosamide following nebulization in the tested dose ranges of 10 to 60 mg NEN (see Fig. S3). Non-compartmental plasma pharmacokinetic parameters of nebulized NEN are summarized in Table II. The estimates of the fixed and random effects of the population pharmacokinetics are reported in Table II and S2. To inform the translatability of absorption kinetics of niclosamide from the sheep model to human, we correlated the observed peak plasma concentration of niclosamide to that reported in the completed Phase 1 trial, in a dose-matched manner [15] (in Backer *et al*., combination of nebulization + intranasal administration was used). Precisely, we correlated the mean C_max_ of cohort 2, 3, 4 of the Phase 1 trial to the mean C_max_ of Group 2, 3 ,4 of this study; in both studies, identical amounts of NEN were nebulized (single doses of 10, 30 and 60 mg). Of note, all three cohorts of the Phase 1 trial included an additional low amount of NEN administered intranasally (3 mg in all three cohorts) whose contribution to the overall plasma exposure is however thought to be negligible and was therefore excluded in the correlation. That is likely because i) plasma exposure is mainly driven by the larger nebulized dose via the lungs having a larger surface area and ii) plasma pharmacokinetics in the Phase 1 trial behaved dose-linear with an increasing nebulized dose while the intranasally administered dose remained identical.

**Table II:** Non-Compartmental pharmacokinetic parameters of niclosamide in plasma and epithelial lining fluid (ELF) following nebulization and oral administration. Absorption rate constant (k_α_) and bioavailability (F) were estimated by population pharmacokinetic modeling. Full list of the parameter estimates of the population pharmacokinetic model for nebulized niclosamide are displayed in Table S2.

Dose Group	Dose (mg/kg)	C_max_ (ng/mL)	T_max_ (min)	AUC_0-t_ (ng/mLxhr)	k_α_	F	T_1/2__α_ (h)	T_1/2__β_ (h)	Elimination T_1/2_ (h)
Plasma									
0.167% NEN Nebulized	0.27	76.6 ± 15.8	27.1 ± 7.9	35.9 ± 11.0	20.9	86%	0.041	0.2	14
0.5% NEN Nebulized	0.76	239.8 ± 63.5	24.3 ± 6.1	96.1 ± 27.8					
1% NEN Nebulized	1.40	577.1 ± 219.8	22.2 ± 8.8	261.4 ±83.4					
Oral Niclosamide	27.88	13.6 ± 6.4	340.0 ± 151.0	17.0 ± 5.6	ND				
Epithelial lining fluid									
0.167% NEN Nebulized	0.27	20306.6 ± 22400.1	ND	12340.4 ± 11757.9	ND				
0.5% NEN Nebulized	0.76	17475.2 ± 17923.4		15345.8 ± 10520.5					
1% NEN Nebulized	1.40	19924.3 ± 26457.3		16144.6 ± 24652.6					
Oral Niclosamide	27.88	34.5 ± 46.6		227.1 ± 165.2					

Mean ± SD where applicable. NEN = niclosamide ethanolamine salt, ND = not determined, C_max_ = maximum concentration, T_max_ = time to peak concentration, AUC = Area under the curve, k_α_ = absorption rate constant (estimated by population pharmacokinetic model). F = Bioavailability (estimated by population pharmacokinetic modeling), T_1/2__α_ = half-life of α-phase. T_1/2__β_ = half-life of β phase.

We found significant correlation in peak niclosamide plasma concentrations in human and sheep within the dose ranges of 10-60 mg, with a correlation coefficient *r* of 0.9987 (Pearson Correlation, p = 0.03) (Fig. 2B). This data suggests that the sheep model describes accurately the absorption kinetics of nebulized NEN in humans.

### Epithelial lining fluid (ELF) concentration following nebulized niclosamide ethanolamine

In contrast to dose-linearity in niclosamide plasma PK, indicating dose-dependent disposition from the lungs after nebulization, the ELF concentration did not behave dose-linear, with the C_max_ and AUC being similar across the three nebulized doses (Fig. 2C and Table II). We further observed that some ELF concentrations within the time-course of sampling could not be calculated due to the measured BALF being below LLOQ. These observations disagreed with basic pharmacokinetic principles and thus may rather reflect technical challenges in the recovery of niclosamide in BALF. Furthermore, significant interindividual variability was observed in each dose group. Thus, an ELF pharmacokinetic model could not be established based on the current data due to the lack of a relationship between dose amount and ELF concentrations and only non-compartmental analysis was used to estimate C_max_ and AUC.

Niclosamide ELF C_max_ and AUC was substantially higher compared to plasma following nebulization, with a partition coefficient Kp_ELF:Plasma_ remaining high over time (Table II); Kp was approximately at 70 (5 min) and 122 (1-2 h) (1% NEN plasma data used for calculation). Niclosamide ELF concentrations rapidly declined within the first hour, followed by a second phase with a slower decline in concentration (Fig. 2C). Although there was no clear dose-linearity in ELF, the general decreasing trend in mean niclosamide concentrations observed across the doses relative to each time point indicates clearance from the lung over time.

### Comparison of lung targeting between nebulization and oral administration

Comparing the mean AUC and apparent C_max_ of niclosamide in ELF following nebulization and a human equivalent 2 g oral dose (1132 mg niclosamide corresponding to 27.88 mg/kg), an increase in lung exposure was demonstrated using the nebulized administration (Fig. 2D). Nebulization of 5% of the oral niclosamide dose resulted in a 71 times higher AUC and 578 times higher C_max,_ in the ELF. Substantially lower pulmonary exposure by the oral route is driven by low plasma exposure and hence only low levels of drug are able to transfer from plasma to the pulmonary compartment: quantifiable ELF niclosamide concentrations in the oral arm were detected in only two sheep (a total of 4 samples) (Table II). This result suggests enhanced lung targeting after nebulized delivery relative to the oral route of administration.

## Discussion

We here present data on the plasma and pulmonary pharmacokinetics of a nebulized NEN solution combined with lung function testing using a well-established sheep model. We showed that absorption and systemic disposition of nebulized NEN is linear and predictable within the dose ranges of 10-60 mg. Importantly, the plasma kinetics in the sheep model described accurately the absorption kinetics of nebulized niclosamide in human in the dose range tested suggesting that the sheep model can be utilized to predict plasma exposure and lung:plasma transition of niclosamide in humans. We further demonstrated that exposure of niclosamide in the ELF is substantially higher following nebulized NEN compared to the human equivalent 2 g oral dose.

Lung function testing in sheep has been used in other preclinical pulmonary PK studies as a valuable tool to assess tolerability of drug aerosols as sheep have reactive airways [17]. Administration of niclosamide via the three tested routes, nebulized, intravenous and oral was well tolerated with no adverse changes observed in lung function. The lung function profile of nebulized niclosamide is well aligned with the Phase 1 data by Backer *et al.* where only mild and transient throat irritation was observed following nebulization of 1% NEN [15]. However, breathing frequency increased following all three nebulized doses in this study which was not seen in the clinic. Inclusion of an “air control” (no treatment) could be helpful in future studies to understand contribution of the inhalation procedure alone to the overall effect. In addition, dose-ranging tolerability studies in irritated airways are warranted to understand the safety of nebulized niclosamide in diseased airways, such as asthma and COPD.

Significant interindividual variability in ELF concentrations were observed in all administration routes. Similar interindividual variability has been observed in other pulmonary PK studies, especially at earlier collection time points where levels can differ up to ~100 fold [16, 18, 21, 22]. A possible explanation for the variability in the serial BAL results could be the sampling procedure used in this sheep model as various lobes were sampled and therefore the return of the BAL wash may have been affected by variations in lung geometry. Furthermore, unlike human BALs where sequential washes are done in a single lobe, with the later washes used to measure epithelial lining fluid levels, only a single wash is used in this sheep model due to the design of multiple serial BALs. Given that a similar degree of interindividual variability has been recorded in previous studies using the same sheep model suggests that variability may be not due a technical difficulty related to our study but reflect a limitation related to the sheep model when using serial BALF sampling. Furthermore, to understand whether this variability is also apparent in human ELF, future human PK studies with nebulized niclosamide should include BALF sampling. This would further help to understand whether the translatability of sheep PK to human is not only valid for plasma exposure, as shown in this study, but also for ELF concentrations.

In addition to variable ELF concentrations, we further observed that measured ELF concentrations did not proportionally increase with dose, and ELF concentrations were below limit of quantification within the time course of sampling. We did investigate whether precipitation of niclosamide in BALF could contribute to these findings by determining niclosamide concentration in the pellet of BALF; however, we did only find negligible concentrations. Further, determination of intracellular lung or lymph concentrations could help to further elucidate transit behavior of niclosamide in the pulmonary compartment, e.g. investigation of a potential reservoir, as it has been done in a previous study with pirfenidone [16]. This assessment was unfortunately not feasible in this study due to practical limitations. However, the observed dose-linearity of plasma exposure in all three nebulized doses indicates a dose-dependent transition of niclosamide from lungs into the plasma (partition coefficient > 1 throughout whole sampling window) suggesting that the observations may reflect technical difficulties with recovering niclosamide in ELF rather than a physiological phenomenon. However, even though technical challenges with the recovery may contribute to the observed effect, the here presented data would be an underestimation of “true” niclosamide concentration in the ELF and therefore can be seen as a conservative estimate.

Despite the lack of dose-proportionality in ELF concentrations, peak concentrations of niclosamide are substantially higher with the nebulized route versus the oral route of administration due to low oral absorption of niclosamide. Low oral bioavailability of 0.5 - 10 % has been described in previous studies in rats and dogs but not in larger animals [7, 23]. We were not able to determine the oral bioavailability in this study as the dataset did not provide enough information to meaningfully model the PK behavior due to lack of plausible and consistent profiles, and high inter- and intraindividual variability. But, plasma concentrations following oral niclosamide were generally lower compared to the limited published data using a similar oral dose in humans as reviewed by Singh *et al.* [3]. Indeed, it has been previously described that for many drugs bioavailability is lower in ruminants compared to humans, but closure of the reticular groove, which we have performed in this study, increases bioavailability and prevents content being held/metabolized in the rumen improving comparability of oral dosing in sheep and humans [24]. However, it remains challenging to contextualize the observed plasma time-course to published oral data as i) no human full PK profile (incl bioavailability) is available, ii) human PK samples were obtained from a few cancer patients with variable concentrations measured and ii) no PK data in a larger animal/ruminant is publicly available. We do believe that the oral dose administered in this study reflects the human dose as the dose was adjusted based on a mg/kg and mg/m^2^ basis according to FDA guidance [25] and the drug was successfully delivered directly to the abomasum circumventing potential metabolism or slower absorption in the rumen [26]. Still, this model may not be an accurate approximation of the absorption of oral niclosamide. Even though we found that this sheep model can be used to predict plasma pharmacokinetics of nebulized niclosamide in humans, further PK studies in humans are needed to understand the translational potential of this model for oral delivery of niclosamide.

## Conclusion

In conclusion, local delivery of niclosamide reaches high concentrations in clinically relevant target tissue for pulmonary conditions and infections, showing a superior pharmacokinetic profile compared to the approved human 2 g oral dose. Thus, niclosamide is a promising treatment candidate for pulmonary diseases, warranting further clinical testing in dose-ranging patient studies.

## Supplementary information

ESM 1 (DOCX 316 kb)
