# Supplementary material for: Single-dose pharmacokinetics and lung function of nebulized niclosamide ethanolamine in sheep
Source: Pharm Res. 2023 Jul 27;40(8):1915–25. doi: 10.1007/s11095-023-03559-0 (PMC10447587; doi:10.1007/s11095-023-03559-0)
Supplement: Supplementary file 1 — (DOCX 316 kb) [file 11095_2023_3559_MOESM1_ESM.docx]

**Supporting Information**

**Single-dose pharmacokinetics and lung function of nebulized niclosamide ethanolamine in sheep**

Anne Weiss^1,2^, Robert J Bischof^3,4,5^, Cornelia B Landersdorfer^5^, Tri-Hung Nguyen^5^, Andrew Davies^6^, Jibriil Ibrahim^4,5^, Paul Wynne^5^, Phillip Wright^5^, Günter Ditzinger^7^, A Bruce Montgomery^8^, Els Meeusen^3,4^, Michelle P McIntosh^5^, Morten OA Sommer^2,7,*^

^1^UNION therapeutics Research Services, Hellerup, Denmark

^2^Novo Nordisk Foundation Center for Biosustainability, Technical University Denmark, Lyngby, Denmark

^3^Allergenix Pty Ltd, Melbourne, Australia

^4^Institute of Innovation, Science and Sustainability, Federation University Australia, Berwick, Australia

^5^Drug Delivery, Disposition and Dynamics, Monash University, Melbourne, Australia

^6^Biomedicine Discovery Institute, Monash University Peninsula Campus, Frankston, Australia

^7^UNION therapeutics, Hellerup, Denmark

^8^ Medina, Washington, USA

*Corresponding author: Morten OA Sommer, [msom@biosustain.dtu.dk](mailto:msom@biosustain.dtu.dk)

**Table S1: Dose information per dose group.**

| **Dose Group** | **Route** | **Concentration [%]** | **Dose [mg]** | **Dose [mg/kg]** | | **Volume [mL]** | **Delivery time (min)** | | **Droplet size (Dv(50)) in µm** | |
| --- | --- | --- | --- | --- | --- | --- | --- | --- | --- | --- |
|  |  |  |  | **Mean** | **SD** |  | **Mean** | **SD** | **Mean** | **SD** |
| 1 | Nebulization | 1% Placebo | NA | NA | | 6 | 24 | 7 | 3.61 | 0.08 |
| 2 | Nebulization | 0.167 % NEN | 10 | 0.271 | 0.035 | 6 | 27 | 5 | 3.38 | 0.19 |
| 3 | Nebulization | 0.5 % NEN | 30 | 0.762 | 0.167 | 6 | 24 | 3 | 2.97 | 0.28 |
| 4 | Nebulization | 1% NEN | 60 | 1.401 | 0.362 | 6 | 25 | 7 | 3.57 | 0.48 |
| 5 | Oral |  | 1132 | 27.88 | 1.64 | 20 | - | - | - | - |
| 6 | Intravenous | 0.1% NEN | 3 | 0.073 | 0.003 | 3 | 30 | - | - | - |

NEN = niclosamide ethanolamine salt. Dv = Volume Median Diameter (of Aerosols). NA = not applicable

**Table S2. Parameter estimates, interindividual variability (IIV) and relative standard error (RSE) expressed as %CV (% coefficient of variation) of the final model for the plasma pharmacokinetics of niclosamide following IV infusion and nebulised administration.**

| **Parameter** | **Symbol** | **Unit** | **Estimate** | **IIV %CV** | **RSE %CV** |
| --- | --- | --- | --- | --- | --- |
| First-order absorption rate constant | k_a_ | h^-1^ | 20.9 | 76 | 84 |
| Bioavailability of nebulised dosing | F_neb_ |  | 85.8% | - | 26 |
| Systemic elimination clearance | CL | L/h | 170 | 34 | 60 |
| Volume of distribution of the central compartment | V1 | L | 15.7 | 71 | 62 |
| Volume of distribution of the shallow peripheral compartment | V2 | L | 10.1 | 28 | 28 |
| Distribution clearance between the central and shallow peripheral compartments | CL_d1_ | L/h | 57.4 | - | 70 |
| Volume of distribution of the deep peripheral compartment | V3 | L | 148 | - | ^a^ |
| Distribution clearance between the central and deep peripheral compartments | CL_d2_ | L/h | 7.47 | - | ^a^ |
| Proportional residual variability | CV_CP_ | % | 25.2 | - | - |
| Additive residual variability | SD_CP_ | ng/mL | 0.26 | - | - |
|  |  |  |  |  |  |

^a^RSE was high (>100% CV) for parameters relating to the deep peripheral compartment, however a two-compartment disposition model (i.e. without a deep peripheral compartment) resulted in a significantly higher objective function (+23.5 at a difference of 2 degrees of freedom) compared to the three-compartment disposition model. Furthermore, three slopes were apparent in the plasma pharmacokinetic profiles following nebulized administration (Fig. S3 1% dose). Simulations from the model to predict pharmacokinetic profiles beyond 3 h for dosing regimens outside the range of those investigated in this study should be interpreted with caution.

**Fig. S1: Fold changes of blood glucose following oral dosing as surrogate for successful closure of reticular groove and direct delivery of drug into the abomasum in sheep.** Mean and Individual glucose plasma concentrations with (blue full circles) and without (clear open circles) CuSO_4_ treatment to sheep (n=5).


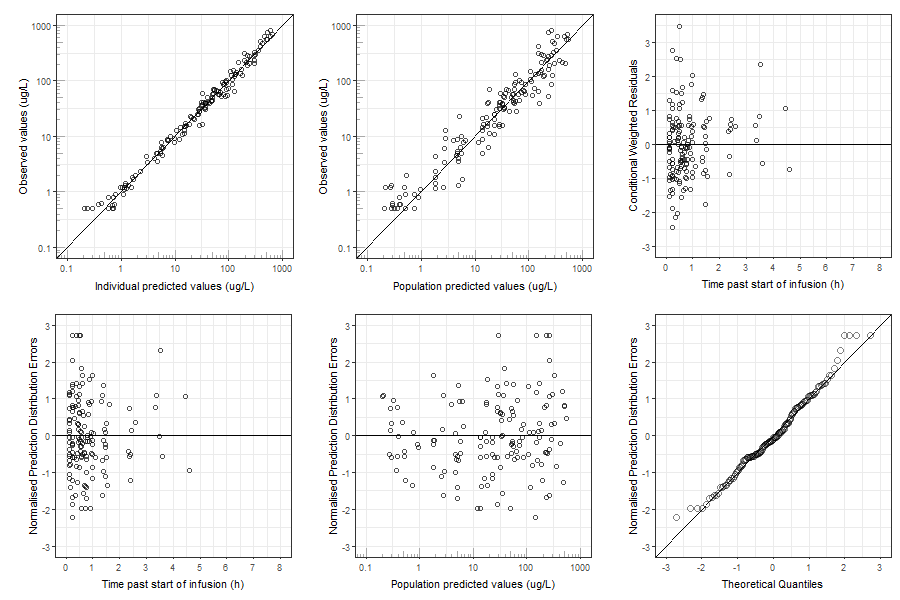


**Fig. S2: Diagnostic plots of the final model for the plasma pharmacokinetics of niclosamide following IV infusion and nebulized dosing.**

| 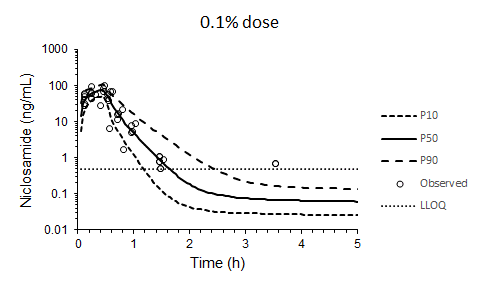 |
| --- |
| 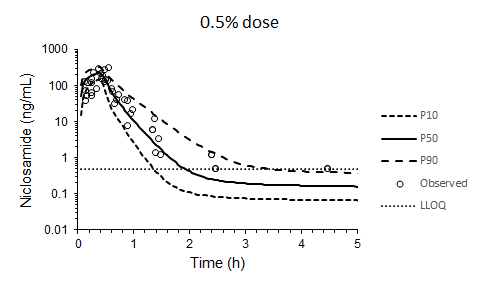 |
| 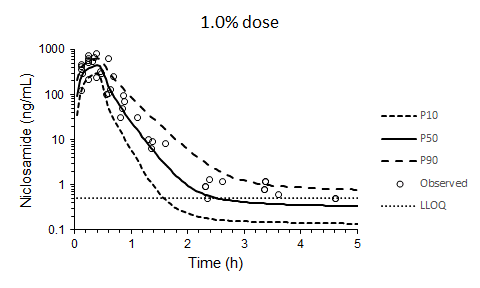 |

**Figure S3. Simulations for nebulized niclosamide stratified by dose level.**

P10: 10^th^ percentile predicted, P50: median predicted, P90: 90^th^ percentile predicted, symbols: observed niclosamide concentrations, LLOQ: lower limit of quantification of the assay.

**Fig. S4: Plasma concentration-time profiles of niclosamide of three administration routes.** Time concentration profiles following nebulized and intravenous administration of niclosamide ethanolamine including all datapoints (A) and following oral administration of niclosamide (B). Data displayed as mean ± SEM on linear (left panel) and semilog scale (right panel). LLOQ = lower limit of quantification. NEN = niclosamide ethanolamine salt.
